# Physiological Arousal Quantifying Perception of Safe and Unsafe Virtual Environments by Older and Younger Adults

**DOI:** 10.3390/s19112447

**Published:** 2019-05-29

**Authors:** Sofia Leite, Miguel S. Dias, Sara Eloy, João Freitas, Sibila Marques, Tiago Pedro, Lázaro Ourique

**Affiliations:** 1 CINTESIS—Center for Health Technology and Services Research, Faculty of Medicine, University of Porto, 4200-450 Porto, Portugal; sofiarsleite@gmail.com; 2 Instituto Universitário de Lisboa (ISCTE-IUL), ISTAR-IUL, 1649-026 Lisboa, Portugal; sibila.marques@iscte-iul.p (M.S.D.); sibila.marques@iscte-iul.pt (S.M.); tiago.miguel.pedro@live.com.pt (T.P.); lazaro.ourique@gmail.com (L.O.); 3 ADENE, Agência para a Energia, 1050-065 Lisboa, Portugal; 4 DefinedCrowd, 1500-399 Lisboa, Portugal; jdcfreitas@live.com.pt

**Keywords:** virtual environment, perception of safety, applied physiology, electrodermal activation sensors, electrocardiogram sensors, architecture, building construction

## Abstract

Physiological arousal has been increasingly applied to monitor exploration (or navigation) of a virtual environment (VE), especially when the VE is designed to evoke an anxiety-related response. The present work aims to evaluate human physiological reactions to safe and unsafe VEs. We compared the effect of the presence of handrails in the VE in two different samples, young and older adults, through self-reports and physiological data: Electrodermal activation (EDA) and electrocardiogram (ECG) sensors. After navigation, self-report questionnaires were administered. We found that the VEs evoked a clearly differentiated perception of safety and unsafety demonstrated through self-reports, with older adults being more discriminative in their responses and reporting a higher sense of presence. In terms of physiological data, the effect of handrails did not provoke significant differences in arousal. Safety was better operationalized by discriminating neutral/non-neutral spaces, where the reaction of older adults was more pronounced than young adults. Results serve as a basis for orienting future experiments in the line of VE and applied physiology usage in the architectural spaces design process. This specific work also provided a basis for the development of applications that integrate virtual reality and applied biofeedback, tapping into mobility and ageing.

## 1. Introduction

Physiological arousal has been increasingly used to monitor human activity during the exploration (or navigation) of a virtual environment (VE). Importantly, using physiological measures for sensing human reactions to a VE requires some considerations [1]. First, the VE must be designed in accordance with what happens in a real environment. Second, the target population using the VE must present some characteristics that overlap with what the VE is expected to evoke. The quality or effectiveness of a VE has been majorly deduced by the degree of presence that it provokes in the user [1], commonly known as the “sense of being in the virtual world”. Because this sense of presence is a subjective condition, it is primarily assessed through subjective measures, such as self-report questionnaires.

Physiological arousal might be an objective indicator of a sense of presence, given that the greater the arousal, the greater the presence [1]. As a matter of fact, using physiological data to quantify presence is relevant, not only for reducing the subjectivity of the sense of presence evaluation, but also for objectively measuring the quality of a VE in evoking the reactions it is expected to evoke. Yet, physiological measures can only be used if they prove reliable, i.e., if results are repeatable across experiments; valid, i.e., if they correlate well with subjective measures of presence taken in self-reports; and sensitive, i.e., if they capture modulations in the VE design. The physiological metrics most commonly used are heart rate (HR), respiration rate (RR), skin conductance level (SCL), skin temperature (ST) and electro encephalographic (EEG) activity [2,3,4,5,6].

The present work fits in this research line of physiological metrics for monitoring navigation in a VE. It is part of a larger set of studies, which long-term goal is to replicate indoor and outdoor architectural spaces in a VE, in a way that they end up eliciting similar physiological responses of arousal as the physical spaces. Hence, we aim to validate the use of biometrics for future comparison of the reactions to indoor and outdoor architectural spaces. In previous work [7], we showed that the VE evoked different reactions that were captured by self-reports and physiological data. We also showed that SCL objectively captured modulations in arousal responses related to “positive” or “negative” emotions, along with the navigation through different architectural spaces in a VE. In the present work, we aim to deepen our understanding of how subjects perceive safety and unsafety in virtual spaces.

Although literature in similar work is not extensive, some studies have been conducted in a height-response eliciting VE. Studies show that architectural design configurations have an impact on the way people behave [8,9], as well as on the stress and anxiety experience of the users of a given space [10,11]. Results are still inconclusive, not only in terms of the physiological measures at use, but also regarding the impact of the VE’s design [12] and the characteristics of the population that is being targeted [2,13]. In these scenarios, a high and significant correlation between presence and change in SCL has been found [14,15]. However, the use of HR change in detriment to the use of SCL change has also been strongly supported [1]. Further work conducted in a flying VE system showed that both HR change and SCL change, display a high correlation with presence, degree of realism and immersion [2,13].

In order to test the reaction to safe and unsafe places, we will quantify the self-reported sensations of “fear of falling”, heights or frustration induced by “unsafe” spaces, and the sensations of relief and safety induced by “safe” spaces. Fear of falling is a psychological phenomenon found at all ages, but which tends to intensify with aging, causing an increasing number of actual falls and cognitive decline, causing reduced mobility, lowered self-confidence and overall decreased quality of life (QoL) [16,17]. Because fear of falling is often associated with increased anxiety during mobility [17], we assume that it can also be captured by an increase in physiological arousal during navigation in VE, especially in unsafe architectural places. As we must consider the conditions associated with mobility in safe and unsafe spaces and how they might affect the individuals’ perception, this study was devised both to evaluate the reliability, validity, and sensitivity of physiological measures in safe and unsafe spaces—namely HR change and SCL, which are two of the most used measures and have yet reached a consensus—and the reaction of young and older people. Importantly, different populations were tested in order to account for the generalizability of our results.

We envision that a discriminating response to the VE navigation through these physiological indicators will serve as preliminary guidance to ensuing studies concerning requirements for a complete simulation of real spaces. Second, we further envision that our results will serve as the grounds for interesting applications that combine the fields of virtual reality (VR) and applied physiology, such as Biofeedback systems. Biofeedback systems are loop systems in which real time information from psychophysiological recordings is provided to the user, who uses it as a means for improving performance [18]. Most of the existing VR systems coupled with Biofeedback are not yet fully integrated. Only recently, a fully integrated system was developed [19]. Besides the efficacy and efficiency of the training process, the authors mention that an adaptable and personalized training environment, which can be conducted remotely at home, are further advantages of integrating both systems. Existing systems focus mainly on anxiety-related disorders, such as generalized-anxiety disorder [19], and other clinical conditions, such as attention deficit hyperactivity disorder, or broader impulsiveness and inattention [20], but there is also interesting and relevant work demonstrating the efficacy of VR training or Biofeedback training targeting age-related conditions associated with anxiety, mobility, and balance [16,21,22,23,24]. In line with this, if our hypotheses are validated in the future, we will be able to integrate VR and Biofeedback and assess the efficacy of a VR-Biofeedback Training system aiming to reduce physiological arousal during navigation in unsafe places. Reducing fear of falling in older adults is also assumed to improve their autonomy and independence in daily activities, increasing their QoL [17]. In fact, those who experience at least one fall admit to having a prolonged fear of falling and, as a result, “25% of these individuals decrease their activity levels”, causing a decline in mobility and independence [25].

### Objectives and Hypothesis

In the present work, our main goal was to detect differences in arousal during navigation in a VE composed of safe and unsafe architectural places to evaluate the validity, sensitivity and reliability of HR change and SCL change. This goal was broken down into specific objectives. First, we aimed to detect differences in how participants perceive the safe and unsafe architectural places of the VE through self-report measures. Second, we aimed to evaluate the extent to which these differences were also captured by physiological measures. Third, we aimed to evaluate whether the physiological modulation of arousal held across participants (reliability) even though they might show different responses. As a secondary objective, we aimed at validating the effectiveness of our semi-immersive virtual space to evoke fear of falling, when compared to an immersive space. Given these objectives, we hypothesize that unsafe places will be subjectively perceived as more threatening (H1) and thus they will induce higher physiological arousal than safe places (H2). We also hypothesize that each physiological measure is correlated, across the two samples being compared—young and older adults (H3), accounting for the reliability of these measures. We further suggest that older adults will perceive unsafe places as more threatening than young adults, which will be manifested by higher physiological arousal for older adults than for younger adults (H4). A final and secondary hypothesis is proposed concerning the effectiveness of our stimuli in terms of its technological properties. Hence, we hypothesize that our semi-immersive VE was as effective in evoking the fear of falling as was the full immersive technology CAVE (Cave Automatic Virtual Environment) at the High Performance Computing Center (from now on HLRS CAVE), used as a reference for comparison (H5). We put this question under testing so that we can demonstrate that our results were obtained in due methodological circumstances.

## 2. Method

A set of three studies was conducted in the same VE scenario. One study was conducted at the High-Performance Computing Center, at Stuttgart University, Germany, in an immersive VR facility from now on called HLRS CAVE (study 1). Two other studies (studies 2 and 3), were conducted at ISCTE—University Institute of Lisbon (ISCTE-IUL), ISTAR-IUL lab, Portugal, in a semi-immersive VR called Pocket CAVE, developed in collaboration between the Digital Living Spaces group of ISTAR-IUL and the Microsoft Language Development Center in Lisbon. Studies 1 and 3 were conducted with a sample of young adults, whereas study 2 was conducted with a sample of older adults. The methodology and procedures adopted for this study were approved by the Ethics Committee of ISCTE-IUL, since they are in accordance with the ethical standards of the responsible committee on human experiments and with the Helsinki Declaration of 1975, as revised in 2000.

### 2.1. Virtual Environment Design

**Figure 1:**
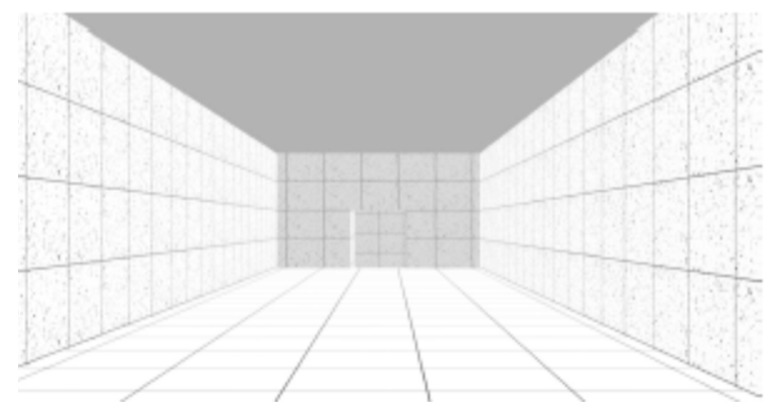
Neutral Rooms (1, 2 and 3).

**Figure 2:**
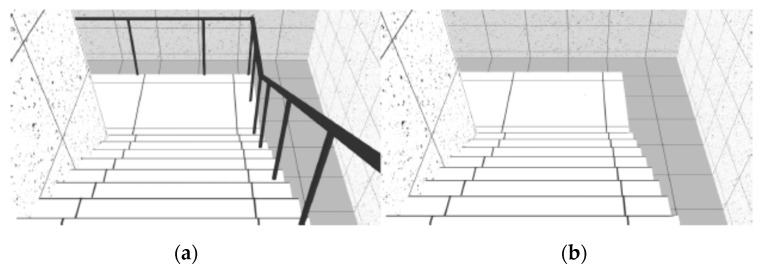
Descending Stairs scenario: (**a**) With handrails; (**b**) without handrails.

**Figure 3:**
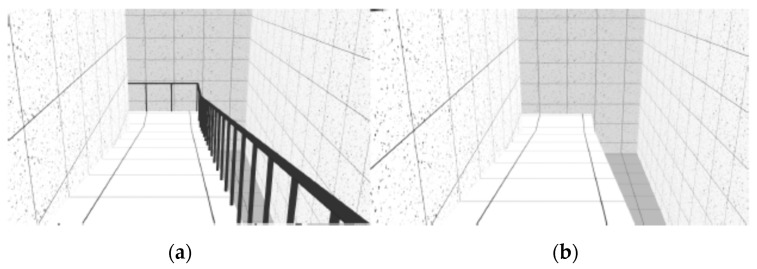
Descending Ramp 1 scenario: (**a**) With handrails; (**b**) without handrails.

**Figure 4:**
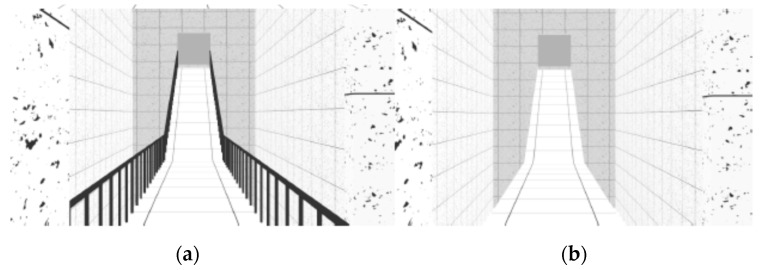
Horizontal plane with Ascending Ramp: (**a**) With handrails; (**b**) without handrails.

The VE was composed of sequenced spaces, like rooms, stairs and ramps, which simulated the interior of a building. As reported in previous studies [26], three neutral rooms were designed with 30 m long by 5.5 m wide (Figure 1). These neutral rooms interchanged with non-neutral spaces, like stairs and ramps. Stairs and ramps were designed according to standard construction regulations. The VE was composed of two flights of descending stairs with 12 steps each (0.28 × 0.18 m) and 1.5 m wide (Figure 2); two flights of descending ramps with 10 m long by 1.5 m wide and 20% slope (Figure 3); one horizontal plane with 1.5 m wide by 10 m long followed by an ascending ramp with the same dimensions and with 40% slope (Figure 4).

It was decided to use a grey concrete texture for all walls that included a rectangular frame simulating the concrete formwork. All floors were grey in color as well and had a square pattern simulating floor tiles. Lighting was done by five light points across the whole sequence of spaces, with the same attenuation factor (linear/quadratic = 0) and falloff distance (25 m).

**Figure 5:**
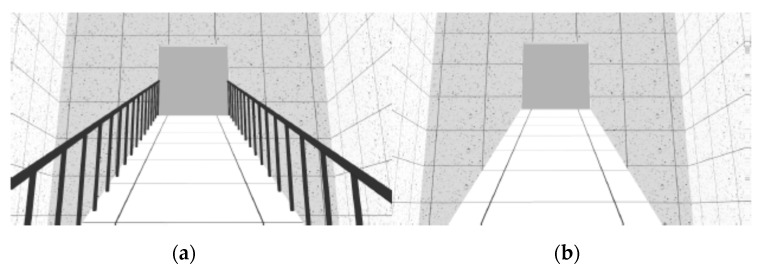
Ascending Ramp: (**a**) With handrails; (**b**) without handrails.

Two geometrically identical virtual models were designed with a difference - established by the presence or absence of handrails in stairs and ramps. The virtual model with handrails was considered a safe environment (left side of Figure 2, Figure 3, Figure 4 and Figure 5) and the one without handrails was considered an unsafe environment (right side of Figure 2, Figure 3, Figure 4 and Figure 5).

A third VE was designed for training trials, composed of two neutral rooms connected in an "L" shape. The same textures and navigational strategy were used for the training environment.

### 2.2. Participants

Eighty-seven subjects in total voluntarily participated in this study. Informed consent was obtained from all participants. As in a previous study conducted by us [26], participants were selected considering the following admission criteria: (i) Meeting age requirements of each study; (ii) Having normal sight or using corrective lenses; (iii) Being able to stand up without support for a long period of time; (iv) Do not have pacemakers; v) Not suffering from claustrophobia; (vi) Not suffering from dizziness.

In study 1 (HLRS CAVE), the sample was composed by 27 subjects recruited from among the members (researchers, interns and staff) of the High-Performance Computing Center at Stuttgart University (mean age = 39.3, SD age = 10.2). 13 navigated the VE with handrails (mean age = 38.7, SD age= 9.8) and 14 navigated the VE without handrails (mean age = 39.9, SD age = 10.9).

In study 2 (Pocket CAVE), the sample was composed of 31 subjects (mean age = 78.5, SD age = 7.2). 12 participants composed the group navigating in the VE with handrails (mean age = 77.8, SD age = 7.8) and 9 the VE without handrails (mean age = 79.2, SD age = 6.7). Participants were recruited from two community centers in Lisbon, as volunteers, and a donation was made to help improve their facilities. Although 31 subjects were recruited and participated in the experiment, only 21 results were considered valid and analyzed, due to lack of consistency of the data collected (some participants did not pass through all the spaces, data were not correctly acquired).

In study 3 (Pocket CAVE), the sample was composed on 29 subjects, all of them undergraduate Architecture students at ISCTE-IUL (mean age = 22.8, SD age = 3.1). 11 navigated the VE with handrails (mean age = 23.1, SD age = 2.9) and 12 navigated the VE without handrails (mean age = 22.4, SD age = 3.3). Again, six participants have been excluded, due to the same reasons stated above.

In all the three studies participants had no relation or knowledge about the study being conducted.

After physiological data pre-processing (explained in Section 3), some subjects were excluded, due to noisy recordings. Hence, for the self-reported data we kept the total number of participants in each study; for EDA data we only considered 18 of 31 recordings in total of older adults and 19 of 29 recordings in total of young adults; and for ECG data we only kept 21 of 31 recordings in total of the older adults and 23 of 29 recordings in total of young adults.

### 2.3. Procedure

For each study, samples were randomly split into two groups: One group was assigned to navigate in the VE with handrails, and the other to navigate in the VE without handrails (handrails condition). The experiment began with the setup of the devices for physiological data collection (ECG and EDA). Afterwards, participants were asked to navigate the training VE until they reported that they had become familiar with the task. Navigation in the experimental VE ensued, with an approximate three-minute duration (mean total duration = 177.7 s; SD total duration = 20.8 s), during which ECG and EDA activity were recorded. The navigation was linear, from the beginning to the end of the circuit, participants would only move forward and never backward, only passing through each space once. There were no interruptions in navigation during the test phase. Subjects crossed the neutral and non-neutral places described above, always following the same seven different spaces-sequence—Neutral Room 1 (Figure 1), Descending Stairs (Figure 2), Neutral Room 2 (Figure 1), Descending Ramp 1 (Figure 3), Descending Ramp 2, Ascending Ramp (Figure 4 and Figure 5), and Neutral Room 3 (Figure 1).

For the present work we did not use the physiological data collected in study 1; nonetheless, we still collected that data to maximize the similarity between the methodologies (the subjects’ circumstances during the experiment). A second reason deals with the fact that this is still an exploratory work in nature, so we might well use this data in future experiments/data analysis procedures.

**Figure 6:**
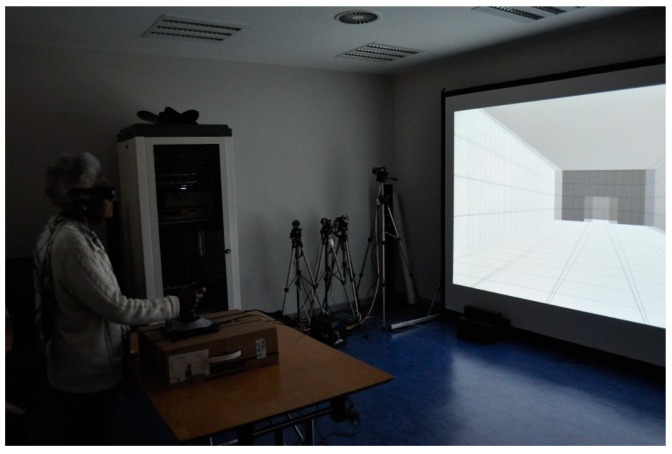
Pocket CAVE developed at ISCTE—University Institute of Lisbon (ISCTE-IUL), ISTAR-IUL lab, Portugal.

For the Pocket CAVE (Figure 6) the virtual model was projected on a 4 m × 3 m screen by a stereoscopic Digital Light Processing (DLP) projector (DepthQ HDs3D-1, Bellevue, WA, US) with 1280 by 720 pixel resolution, and visualized with active glasses (nVIDIA® 3DVision(TM)2, Santa Clara, CA, US). The observation distance (i.e. the distance between the observers’ eyes and the screen) was 3.50 m. The virtual camera had 45° of horizontal Field of View (FOV) and 33° of vertical FOV, approximately. Participants navigated in the VE in a fixed path and used a joystick (Logitech Extreme 3D Pro, Lausanne, Switzerland) with a constant displacement speed of 0.82 m/s [27] for moving forward or to stop. We used the CAVE Hollowspace software system [28] fully developed and maintained in-house by our research team.

**Figure 7:**
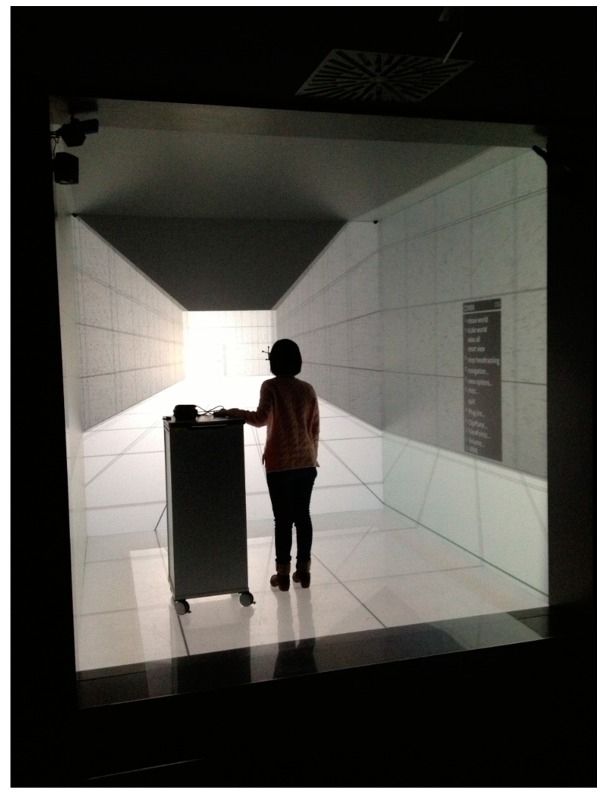
HLRS CAVE developed at the High Performance Computing Center, at Stuttgart University, Germany.

For the HLRS CAVE study (Figure 7), the virtual model was presented on five surfaces, forming a cube of 2.7 m edge. In this CAVE images are back projected on each of the five walls with top and bottom projections being done from different floor levels. DLP projectors offer a resolution of 1920 by 1200 pixels. Participants used 3-D glasses with head tracking (ART tracking systems) enabling them to orient images so that they appear naturally to the human eye. Hence, four cameras at the corners of the ceiling track the users’ glasses and input device to keep virtual environments in control of researchers (HLRS site). The observation distance (i.e. the distance between the observers’ eyes and the screen) was circa 2.0 m. Participants navigated in the VE in a fixed path with a constant displacement speed of 0.82 m/s [27] for moving forward only. In HLRS CAVE, the COVISE software was used.

Finally, after navigation, participants were asked to answer three questionnaires (Supplementary Materials). Two were administered to assess the degree of “Sense of Presence”, the Slater, Usoh, and Steed (SUS) scale [29] and the Witmer and Singer (W&S) scale [30]. A third questionnaire was drawn up to evaluate how participants perceived the space they navigated (with or without handrails), by assessing Perceived Safety, Perceived Fear and Anxiety in each of the non-neutral spaces, through a 7-point Likert-scale. It was administered to assess the degree of “Fear of Falling” that the VE navigation triggered in the subjects and from now on this third questionnaire will be referred to as the “Fear of Falling” questionnaire. Preliminary analyses revealed good psychometric qualities of the Fear of Falling questions in each of the non-neutral spaces for both younger and older participants (Chronbach alphas varied between 0.65 and 0.86). However, the results for the SUS and W&S scales were not so consistent. Hence, we analyzed the results considering the responses to each individual item.

### 2.4. Physiological Data Collection

In the Pocket CAVE studies (studies 2 and 3), physiological data were collected using the BioPLUX research system (Fluxion Biosciences, Inc, Alameda, CA, US), version 1.2 (2010). BioPLUX research system has eight channels. In each channel, specific physiological data acquisition sensors can be connected. The signal is acquired at a sampling frequency of 1000 Hz, with 12-bit resolution, and sent to the computer through Bluetooth communication with a range of 100 m. ECG sensors capture the voltage of the heart’s electrical activity of with a range of +/−1.5 mV and a bandwidth between 0.5 and 100 Hz, with an input impedance above 100 GOhm and with a common-mode rejection ratio of 100 dB. EDA sensors capture skin conductance data within a range of 0–13 µs and a bandwidth between 0–3 Hz, with an input impedance above 1 GOhm and with a common-mode rejection ratio of 100 dB.

In HLRS CAVE physiological data were collected using a RaspberryPi Embedded System that recorded data from the sensors and sent it to the CAVE MasterPC via Wifi. The RaspberryPi ran a version of Debian Linux and was programmed in C to perform the task. e-Health Platform sensors were used to measure HR and to measure it we used the e-health Platform with SPO2 sensor and an analog-digital-converter on an ABIO-Card to make the signal "readable" for the Raspberry Pi.

### 2.5. Physiological Data Processing and Feature Extraction

Physiological data processing was conducted in MatLab, version R2013a. ECG data were high-pass filtered at 0.05 Hz. In order to proceed with the detection of R peaks, the minimum peak distance and the minimum peak height were calculated. The minimum peak distance was calculated based on an arbitrary maximum HR of 150 bpm (beats per minute). Based on this criterion, peaks were detected for a subset of the signal. The average peak height was then calculated, and the minimum peak height was defined as 2/3 of the average peak height. Furthermore, we should add that these criteria were employed for R peak detection based on a backwards elimination process. The minimum peak height was only kept as a criterion for peak detection as long as the total number of peaks was above a certain threshold, calculated as a function of peak distances. After R peak detection was completed, HR was calculated, and the resulting values were standardized in reference to the baseline. Baseline was calculated as the average value of HR from the beginning of the experiment until the end of the first neutral room.

EDA data were visually inspected for removal of noisy subsets of the signal. For the determination of the tonic measure SCL, the signal was decimated for 1 Hz and then low pass filtered at 0.4 Hz. Finally, the resulting values were normalized in order to remove inter-individual variability.

After the physiological features HR and SCL were extracted from ECG and EDA signals, respectively, we defined a sliding window of 10 samples, and calculated the change in arousal between the last and the first sample of each window, for the entire length of the resulting signal of each VE space, from which we computed the features used in further statistical analysis—HR change and SCL change. This operation was conducted to deal with the problem of short navigation in each VE space.

Finally, the average value of each of these resulting features—HR change and SCL change—was calculated for each space of the VE. For this operation, a window of 10 samples (two samples before the entrance in the space and eight afterwards) was considered. Physiological data matrices for statistical analyses were composed of eight columns and N rows: One of these columns identified the group of the subjects—with or without handrails—and the remaining seven represented the arousal in each VE space; N was the number of subjects.

### 2.6. Experimental Design and Statistics Rationale

The present work entailed between-subject and within-subject analyses. Between-subject analyses were conducted to evaluate differences between safe and unsafe spaces in VE (handrails effect) (H1 and H2), to evaluate differences between samples of young and older adults (sample effect) (H4) and to evaluate the effectiveness of fear of falling evocation given the semi-immersive proprieties of the Pocket CAVE when compared to the HLRS CAVE (H5). During the data analysis procedures, we decided to deeply explore the captured biometric data to better understand what effects have been captured in terms of physiological arousal; hence, a within-subject analysis was also conducted to explore differences between spaces in the same VE (space effect). We assumed that neutral rooms would be considered safe spaces whereas non-neutral elements, like stairs and ramps, would be considered unsafe spaces, irrespectively of the presence or absence of handrails. These procedures were conducted posteriori, which explains why they were not hypothesized. Finally, a correlation was performed to evaluate the reliability of both measures across the studies with the young and older people in a semi-immersive Pocket CAVE (H3).

For testing hypotheses 1 to 4, we used the data collected at ISCTE—University Institute of Lisbon (ISCTE-IUL), ISTAR-IUL lab, Portugal, with young and older adults (studies 2 and 3), during navigation in the Pocket CAVE. For testing the final and secondary Hypothesis 5, we used the self-report data collected with young adults at ISCTE—University Institute of Lisbon (ISCTE-IUL), ISTAR-IUL lab, Portugal, and compared it against the self-report data collected at the High-Performance Computing Center, at Stuttgart University, with young adults, too.

Non-parametric tests were conducted, due to the non-normal distribution of the physiological data and the ordinal nature of the self-report data. For between-subject analyses, we performed Mann-Whitney tests, which is the non-parametric equivalent of the t-test. The test statistic U of the Mann-Whitney approach consists of two simple steps. First, every value of group A is compared to every value of group B; second, the number of times the value in A is higher than the value in B is summed up, and vice-versa; U is the smallest of this summation. The closest U is from 0, the more the two groups diverge, the higher the significance (the alternative hypothesis can then be accepted). The closest U is from half the value of A × B, the lower the difference between the groups, the lower the significance (the null hypothesis remains true). For within-subject analyses, we conducted Friedman’s ANOVA, which is assumed as the equivalent non-parametric of ANOVA for Repeated Measures. The test statistic of Friedman’s Chi Squared (X2) is a Chi Squared distribution, calculated in accordance with the number of measures per subject (in our case, to the number of spaces under consideration): k(k + 1)/12. The numerator will be the summation of the squared deviations of each group means to the expected mean if the null hypothesis would remain true. Post-hoc analyzes by conducting the Wilcoxon sign-rank tests, the non-parametric equivalent of the paired sample t-test. In order to analyze the correlation of each physiological measure across studies, the Spearman’s Rank Correlation Coefficient, or Spearman’s rho, was calculated.

Statistical significance was considered at a 95% confidence level, except for contrasts, where Bonferroni procedures were performed to correct the value of significance (value of significance / # of comparisons = corrected value of significance).

## 3. Results

First, we tested whether there were differences in the subjective perception of safe and unsafe spaces in the studies conducted at ISCTE with young and older adults (studies 2 and 3). For the following hypotheses, we merged both groups (young and older adults) as we were interested in evaluating the effect of the handrails in the subjective and objective perception of space safety. The self-report data collected in the “Fear of Falling” questionnaire allowed us to discriminate the perception of safe and unsafe spaces, characterized by the presence or absence of handrails, respectively. Regardless of the participants’ age, unsafe spaces provoked a higher perceived fear, higher anxiety and lower perceived safety than safe spaces (Table 1). Besides low values of p indicating significance, the values of U are also congruent, with U < [(N(safe) × N(unsafe))/2] = 449.5.

Second, we proceeded to test whether these differences in subjective perception were extended to a more automatic level of information processing that could be captured by physiological activity. Yet, although the effect of handrails was pronounced in self-report data, these differences were not held for either of the physiological measures. Nonetheless, because our VE was developed to evoke an anxiety-related response especially for the older adults’ group, we introduced a new hypothesis that aimed to evaluate the reaction to neutral (safe) and non-neutral rooms (unsafe) rather than dealing with the effect of handrails. We found that physiological data tended to discriminate neutral and non-neutral spaces, which correspond to safe and unsafe spaces, respectively (Table 2).

For SCL change, Neutral Room 2 and Descending Stairs were central events for the emergence of differences. Descriptive statistics (Table 3) show: A peak in Neutral Room 1 that afterwards decreased in Descending Stairs; a second peak in Neutral Room 2 that persisted during Descending Ramp 1 and started decreasing in Descending Ramp 2; and a final elevation in Ascending Ramp that decreased afterwards in Neutral Room 3. The presence of peaks in neutral rooms will be discussed ahead. For HR change, we also found differences between spaces, irrespectively of the sample (Table 2). These differences were calculated excluding Neutral Room 3 because of noisy data. As shown in descriptive statistics below (Table 3), there was a peak for Descending Stairs, as expected, that emerged after the lowest arousal elicited by Neutral Room 1. Arousal started decreasing afterwards along with the remaining spaces.

**Figure 8:**
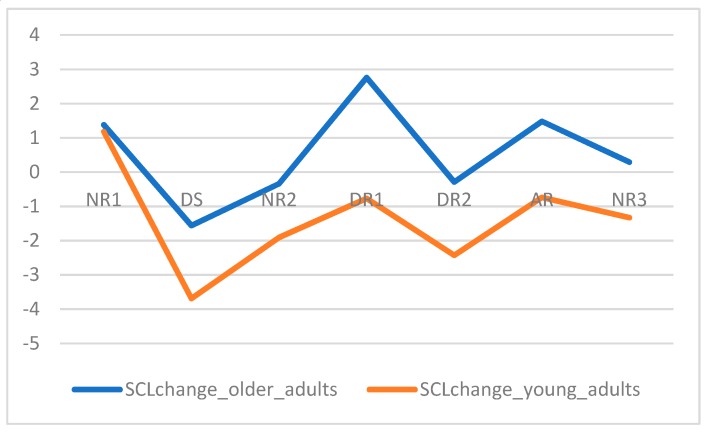
(Mean) SCL change, measured in µS, for studies 2 and 3.

**Figure 9:**
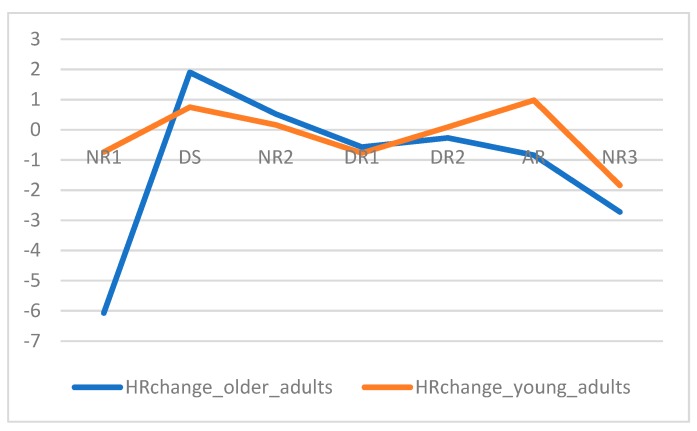
(Mean) HR Acceleration, measured in bpms, for studies 2 and 3.

Third, we tested how reliable both measures were, i.e., to what extent the modulation of physiological measures was correlated across studies 2 and 3. The modulation of physiological measures was taken as the sequence of mean activation per event. Hence, we correlated the values of mean SCL change per event in young adults’ experiment with the values of mean SCL change per event in older adults’ experiment (Figure 8); we did the same for HR change (Figure 9). We found that both SCL change and HR change were positively and significantly correlated, at ρ = 0.82 and ρ = 0.86, respectively.

Up until this point, the results concern differences irrespectively of the age of the participants. From this point on, we will segregate the samples. The idea is not so much to compare groups of different ages to evaluate the effect of age, but to gather data about the generalizability of our results when applying the same experiment to different samples.

First, for the self-report data collected in the “Fear of Falling” questionnaire, we found a sample effect in two of the seven spaces. Young adults showed higher perceived fear and anxiety than older adults for the non-neutral spaces Descending Stairs and Ascending Ramp (Table 4). Besides low values of p indicating significance, the values of U are also congruent, with U < [(N(safe) × N(unsafe))/2] = 449.5.

We tested whether these differences could have been a consequence of differences in sense of presence between both groups of participants; however, older adults consistently reported a higher sense of presence than young adults (Table 5), which held independently of the presence or absence of handrails. Besides low values of p indicating significance, the values of U are also congruent, with U < [(N(safe) × N(unsafe))/2] = 449.5.

In order to disambiguate the source of these results, we scanned the self-report data and suggest that young adults relied more on central scores than older adults. In order to confirm this suggestion, we assessed the interaction between the sample and the handrails effect and found that participants responded to the questionnaires with differentiated patterns for safe and unsafe conditions. For instance, older adults reported significantly higher scores than young adults for perceived safety and significantly lower scores for perceived fear and anxiety (Table 6). It became clear that young adults concentrated their responses in more central scores than older adults, who described their experience through more dispersed or discriminative scores. In other words, although the statistic tests still manifest differences in opposite directions, we believe we could demonstrate that the source of these unexpected results was due to a significantly higher variability in the older adults’ data than in young adults’ data.

In terms of objective measures, we found that physiological data tended to confirm sample effects, although the results were not consistent across measures. For SCL change, a sample effect was verified for Descending Stairs and Descending Ramp 1 (Table 7), with older adults showing increased arousal than young adults. This matches self-reports, with older adults reporting a higher perceived fear, higher anxiety, and lower perceived safety than young adults. For HR change, no sample effects were found. Besides low values of p indicating significance, the values of U are also congruent, with U < [(N(young adults) × N(older adults))/2] = 171.

We proceeded to test the interaction effects between the sample and both the handrails (safe vs. unsafe conditions) and space effect. For SCL change, crossing the sample with the handrails effect, we obtained that for the safe condition (U = 19.00) in Descending Ramp 1, older people displayed higher activation (mean = 4.05; SD = 4.36) than young adults (mean = −1.50; SD = 5.09). For the unsafe condition, no differences were found between groups. For HR change, we found an interaction effect for the unsafe condition for Descending Ramp 1 also (U = 19.00), with older adults showing further decreased activation (mean = −3.09; SD = 4.64) than young adults (mean = 0.93; SD = 2.20), which again was not expected. When considering each sample separately, we still did not find any differences between the presence and absence or handrails. We proceeded to evaluate the space effect for each sample separately. Statistically significant differences in the modulation of HR physiological activity throughout the spaces of the VE were majorly found for older adults (Table 8), this modulation showing the pattern previously found for the space effect irrespectively of the samples.

Finally, the above-mentioned results were obtained during navigation in a semi-immersive VE. Hence, as explained in H5, we tested whether the reaction to the VE we designed – presented in the semi-immersive Pocket CAVE or in the full-immersive HLRS CAVE – was effective in evoking the fear of falling given the different immersion characteristics. We found no differences in almost all items of “Sense of Presence” and “Fear of Falling” questionnaires, except two. Although the median scores were very close to each other, statistically significant differences were found between participants navigating in the HLRS CAVE and the Pocket CAVE, as regards control devices awareness and sense of presence (Table 9). Besides low values of p indicating significance, the values of U are also congruent, with U < [(N(ISCTE) × N(HLRS))/2] = 391.5.

## 4. Discussion

Our envisioned goal is to develop virtual spaces that simulate real spaces and likely elicit the same reactions in the user. This is important and innovative as it might allow that VR and Biofeedback is applied to Architecture and Building Construction design processes. Hence, we must explore the impact of VE, as well as the impact of the participants’ characteristics in perceiving the VE [1]. In this specific work, the link between the reaction to unsafe places and fear of falling in older adults was explored. It further inspired us to the development of applications that combine both fields of VR and Applied Physiology, such as Biofeedback systems. Recent work in this domain shows how effective this kind of applications might be in training several conditions associated with anxiety and ageing, improving the QoL of older people [11,13,16,17,18,19].

In the present work, it was our objective to demonstrate that differences in arousal emerge when participants navigate through safe and unsafe VEs. We devised an experiment for first testing the effectiveness of the VE in evoking different perceptions of safeness throughout different spaces, following necessary requirements [1]. Afterwards, we tested the validity, sensitivity and reliability of physiological measures to quantify the reaction to those spaces, using SCL change and HR change. We quantified the response of young and older adults to understand how the perception of safeness of the VE could be related to the Fear of Falling, a condition that is present at all ages, but which mostly affects mobility in older people [16,17].

Safe and unsafe spaces were primarily characterized by the presence or absence of handrails, respectively. Self-report data concerning perceived safety, perceived fear and anxiety showed that this manipulation was indeed effective for producing different subjective perceptions of safeness during navigation, thus confirming our first hypothesis. However, these discriminated subjective perceptions of the presence or absence of handrails, were not demonstrated by either of the physiological measures (SCL change and HR change). This may be interpreted as handrails being an architectural element that people learn to associate with safety, the meaning of which is possibly encoded by means of top-down controlled mechanisms - captured in self-reports - rather than in automatic and involuntary bottom-up mechanisms, obtained in biometric data. The fact that these differences in physiological arousal did not entirely match the robustness of self-report data, aligns well with fear of falling being primarily defined as a psychological experience. Hence, according to the definition of the validity of physiological measures within the scope of VR experiments [1], we conclude that either SCL change or HR change were valid measures, as they did not capture the effect of the handrails found for self-reports. However, we still hold the suggestion that future experiments should operationalize safety in a different way (with a different architectural element), that elicits a more automatic response. In other words, SCL change and HR change might have not proven valid, due to a poor operationalization of safety.

We proceeded to test a secondary manipulation of safety that was introduced afterwards concerning the sequence of neutral and non-neutral spaces. We found the differences in arousal interesting, along with the navigation in the VE, with differentiated patterns for both SCL change and HR change. For SCL change, we found an initial peak in Neutral Room 1. Although we were not expecting to detect arousal in a neutral room, this result is in line with previous studies showing that SCL is affected by the novelty effect [13]; this was verified even though we included a training phase that lasted until the subject reported being accommodated to the task. Furthermore, we also conclude that the SCL recordings captured modulations, along with the spaces (a space or event effect), if we consider the fact that SCL is a slow-moving parameter that resulted in delayed peaks—SCL change positive peaks usually occurred in the space adjacent to the space that must have elicited the arousal. Latencies of SCL change are assumed to be in the range of 1 or 2 s [31]. For HR change, although it initiated as expected, reaching the first peak in Descending Stairs, it subsequently decreased, along with navigation in the remaining spaces, whether neutral or non-neutral. We assume that the habituation effect possibly impacted on HR which leads us to conclude that SCL change might be a more sensitive physiological measure for the purpose defined herein. In line with previous findings, SCL change captured the novelty and space (or event) effects [14,15]. Although the validity and sensitivity of these measures were not clear enough in terms of reliability, our third hypothesis was confirmed, showing that both measures are reliable and allow for/provide/ express/produce a positive correlation across experiments irrespectively of the presence and absence of handrails, and irrespectively of age differences.

Still regarding the validity and sensitivity of the physiological measures, we suggest that in future studies the time spent crossing spaces be extended (increasing the length of the concerned spaces) to capture more pronounced physiological response. This is suggested to benefit both measures. For SCL change, these reformulations in the VE might be a solution for solving the novelty effect problem (for the first neutral room) and consequently for having all neutral rooms acting as stabilizers of arousal, as well as for minimizing the delay of the SCL response—if VE spaces are more extended, we will be able to detect a physiological response within the correct window. For HR change - given that this metric was reliable across studies - it remains an open question whether these reformulations would also trigger higher amplitudes in HR change in a way that differences among spaces are identified. This question also remains open because previous findings reported the validity of HR change in measuring user experience and sense of presence in anxiety-evoking VE [1,2,12,13]. It is further relevant to mention the high variance in physiological data, which certainly introduced noise in data analysis and its interpretation. We assume that the above-mentioned reformulations will also play an important role in decreasing variance, for obvious reasons. Furthermore, in order to test the validity of physiological measures in respect to the space effect, a questionnaire of space perception concerning the space effect should be constructed and administered, as well. Finally, these reformulations also require that safety is operationalized in a different way, yet to be determined.

We proceeded by evaluating if there was a differentiated reaction between young and older adults along navigation in the different spaces. [16] In terms of self-report data, unexpected results were obtained, with young adults reporting less perceived safety and higher perceived fear and anxiety than older adults in two of the seven spaces, even though older adults reported a higher sense of presence than young adults. After disambiguation, we concluded that results suffered the interference of increased variability in older adults’ responses when compared to young adults. This increased variability in the older sample is in line with previous studies showing similar types of effects [32], and may well occur due to a higher sense of presence in this group. In other words, because older people perceive a higher immersion [11], they are more discriminative in their responses. The increased sense of presence showed by older adults might be associated with the fact that this group is less used to operate with technology and, thus, more permeable to it [17]. In terms of the physiological data, one can begin to raise the question of whether comparing the response of different aged groups is valid. Yet, if age (and its associated physiological events) had taken over the effect of other variables, significant differences between groups would have been found across all experimental conditions, which was not the case. Hence, we proceeded to interpreting results. We also found that differences between spaces were more pronounced for the older people group. We found that the space effects possibly resulted from the heightened sensitivity of older adults to the VE, as we did not find many differences between spaces for the young adults. This is not only in line with older people reporting a higher sense of presence, but also in line with Fear of Falling emerging in older adults, who reported more discriminative answers to the questionnaire and who potentially display higher levels of anxiety (manifested through higher arousal) when facing unsafe/non-neutral spaces [12,13]. Hence, we went further in evaluating the interaction between sample and handrails effects. We did not find a consistent pattern of responses. One the one hand, we put this inconsistency down to possible interference of high variance in the data. On the other hand, however, it was clear that Descending Ramp 1 is a space frequently associated with significant differences in arousal for both measures in both safe and unsafe conditions. This is worth exploring in future studies using the same VE.

Finally, we consider that this work was successful in giving preliminary and necessary steps towards designing a VE that evokes a differentiated perception of safety and unsafety, which can be measured through physiological activity. Our first operationalization of safety/unsafety did not prove effective, but the design of the VE did. Furthermore, as was confirmed by the final and secondary hypothesis, the VE held effective in evoking fear of falling in both a semi-immersive Pocket CAVE and in a full-immersive HLRS CAVE. In terms of using physiological measures, we found that both measures are reliable, but further improvements in the methodology are needed to increase their validity and sensitivity. This study also showed that SCL change was prone to capture the novelty effect; that HR change possibly captured habituation effects; and that physiological measurement was more pronounced for older adults, aligning well with the definition of Fear of Falling. The results also allowed for the identification of possible sources of noise to improve future experiments.

### Limitations

The main limitations of the present work have been discussed earlier, namely, reformulations in the VE such that more time is spent in each separate space, and the operationalization of safety, by thinking of including in the VE, different architectural elements other than handrails.

In terms of self-report data analysis, although the SUS and W&S scales are robust measures used in the literature, our analyses revealed some limitations in their reliability. Hence, these self-report results should be taken with caution and should be further explored in future studies using subjective evaluations of the navigation experience. In terms of physiological data, the fact that some collections were noisy and useless, could have compromised the robustness of our samples.

## 5. Conclusions

In line with previous findings [7,26], this work allowed to conclude that our VE was effective in evoking differentiated reactions for safe and unsafe spaces, irrespectively of the immersive properties of the CAVE in which it was presented. It also allowed us to determine that, under the present circumstances, HR change and SCL change are reliable physiological measures, but the reaction to different spaces along the VE can only be objectively measured through SCL change. Future work is then needed to improve its validity and sensitivity, and obviously, future work should also be oriented towards achieving valid and sensitive HR recordings. Our results also allowed us to draw a link between older adults’ physiological reaction and fear of falling by comparison between samples of older and young adults. This is relevant for the future development of applications that integrate VR and Applied Biofeedback for the purposes of performance optimization and assist in architectural design. Nowadays, there is a high prevalence of VR systems and Biofeedback systems for training people to improve performance in specific areas of their needs [16,18,21,22,23,24]. Yet to this day, little work has been conducted to integrate both modalities (VR and Biofeedback) in one single training system [19]. This integration accounts for positive results in terms of efficacy and efficiency of training, and can be conducted remotely, on an adaptable and personalized basis [19]. Within the larger scope in which this experiment was devised—concerning the future development of VE architectural spaces simulating real environments—this work was fruitful in indicating that some architectural elements, like handrails, are discriminative of a conscious perception of space, but not discriminative in terms of an involuntary automatic perception eventually captured by physiological data. However, results also point towards the effectiveness of a VE in producing a modulation of arousal if the spaces that compose it are considered the sources of safety/unsafety perceptions. Moreover, this work was fruitful in uncovering the effects that are majorly captured by the physiological measurements (novelty and habituation effects) and, thus, in reformulating the methodology for future experiments. Although these results open several doors to future studies, they also provide knowledge about the limitations that we will encounter from now on. Future experiments are already being planned at our research center at ISCTE—University Institute of Lisbon (ISCTE-IUL), ISTAR-IUL VR lab, Portugal, to replicate in an indoor VE space experiments carried out in an outdoor real space to. Data analysis is being conducted to explore users’ differential physiological reaction to both indoor and outdoor spaces in a simple exploration task. We believe that more robust conclusions will be drawn after a composite analysis of both studies.

## Figures and Tables

**Table 1:** Condition effect for the perceived fear of falling.

	Fear of Falling Questionnaire								
	Descending Stairs (Desc_Stairs)			Descending Ramp (Desc_Ramp)			Ascending Ramp (Asc_Ramp)		
	Safe	Fear	Anxiety	Safe	Fear	Anxiety	Safe	Fear	Anxiety
**Safe** (With handrails)	mdn = 5 sd = 1.61	mdn = 2 sd = 1.20	mdn = 2 sd = 1.52	mdn = 5 sd = 1.71	mdn = 1 sd = 1.24	mdn = 2 sd = 1.26	mdn = 4 sd = 1.91	mdn = 2 sd = 1.82	mdn = 3 sd = 1.81
**Unsafe** (Without Handrails)	mdn = 3 sd = 1.55	mdn = 4 sd = 1.50	mdn = 4 sd = 1.69	mdn = 2 sd = 1.24	mdn = 3 sd = 1.68	mdn = 3 sd = 1.64	mdn = 2 sd = 1.59	mdn = 4 sd = 1.73	mdn = 4 sd = 1.88
**Mann-Witney**	U = 175.5	U = 223.0	-	U = 117.5	U = 219.5	U = 288.5	U = 203.5	U = 284.5	U = 300.5

Columns in the first level represent the non-neutral spaces for which there was a handrails effect and in the second level represent the items of the scales for which the handrails effect was true for that space; rows represent the median (mdn) and standard deviations (sd) for each sample and the value of the non-parametric Mann-Witney test (U), respectively.

**Table 2:** Space effect for each physiological measure, irrespectively of the sample.

SCL Change	HR Change
X^2^(6) = 17.05	X^2^(5) = 19.83
NR2—Desc_Stairs	NR1—Desc_Stairs
NR3—Desc_Stairs	NR1—Desc_Ramps
NR2—NR3	NR1—Asc_Ramp
	NR1—NR2

The first row represents the value of the Chi Squared test for differences between neutral and non-neutral spaces. The following rows represent pairs of spaces for which significant differences were found. Descending Stairs (Desc_Stairs), Descending Ramp (Desc_Ramp), Ascending Ramp (Asc_Ramp), Neutral Room (NR).

**Table 3:** Descriptive statistics of SCL change and HR change for each neutral and non-neutral spaces, irrespectively of the sample.

		NR1	Desc_Stairs	NR2	Desc_Ramp1	Desc_Ramp2	Asc_Ramp	NR3
SCL change	mean	1.91	−1.35	0.56	0.58	−0.29	0.40	−0.88
	St.dev.	8.91	7.67	9.87	4.83	5.39	5.36	4.68
HR change	mean	−3.17	1.26	0.33	−0.47	−0.39	−0.10	
	st.dev.	12.69	3.96	7.14	4.24	3.67	10.19	

Descending Stairs (Desc_Stairs), Descending Ramp (Desc_Ramp), Ascending Ramp (Asc_Ramp), Neutral Room (NR).

**Table 4:** Sample effect in the perceived fear of falling.

	Fear of Falling Questionnaire		
	Desc_Stairs	Asc_Ramp	
	Anxiety	Fear	Anxiety
Young adults	mdn = 4 sd = 1.35	mdn = 3 sd = 1.68	mdn = 4 sd = 1.84
Older adults	mdn = 2 sd = 1.73	mdn = 3 sd = 2.01	mdn = 3 sd = 1.96
Mann-Witney	U = 282.0	U = 292.0	U = 257.5

Columns in the first level represent the non-neutral spaces for which there was a sample effect and in the second level represent the items of the scales for which the sample effect was true for that space; rows represent the median (mdn) and standard deviations (sd) for each sample and the value of the non-parametric Mann-Witney test (U), respectively. Descending Stairs (Desc_Stairs), Descending Ramp (Desc_Ramp), Ascending Ramp (Asc_Ramp), Neutral Room (NR).

**Table 5:** Sample effect in the self-reported sense of presence, in studies 2 and 3.

	SUS Presence Scale		W&S Scale		
	Sense of Presence	VE as Reality	Simplicity	Awareness of Devices	Distractibility of Devices
Young adults	mdn = 3 sd = 1.21	mdn = 3 sd = 1.57	mdn = 4 sd = 1.58	mdn = 5 sd = 1.68	mdn = 2 sd = 1.60
Older adults	mdn = 6 sd = 2.15	mdn = 6 sd = 2.42	mdn = 7 sd = 1.62	mdn = 1 sd = 1.66	mdn = 1 sd = 0.75
Mann-Witney	U = 223.5	U = 253.0	U = 154.0	U = 104.0	U = 223.5

Columns represent the items of the scales for which there was a sample effect; rows represent the median (mdn) and standard deviations (sd) for each sample and the value of the non-parametric Mann-Witney test (U), respectively.

**Table 6:** Descriptive statistics discriminating the handrails condition for each sample in self-report questionnaires (studies 2 and 3).

Older Adults				Young Adults			
Safe Condition		Unsafe Condition		Safe Condition		Unsafe Condition	
mdn	sd	mdn	sd	mdn	sd	mdn	sd
5.5	1.49	3	1.79	5	1.63	2	1.31
1	0.48	4	1.7	3	1.13	3	1.23
1.5	0.79	3	2.06	4	1.45	4	1.25
5.5	1.58	2	1.33	4	1.8	2	1.14
1	0.45	3	2.1	2	1.51	3	1.11
1	0.81	3	2.04	2	1.45	3	1.14
4.5	1.99	2	1.76	4	1.87	2	1.45
1	1.78	4	1.88	3	1.74	3.5	1.63
2	1.33	5	1.95	5	1.88	4	1.86
6	1.71	3	2.02	4	1.87	2.5	1.09
1	0.81	3	1.71	3	1.99	3.5	1.4
1	1.15	3	2.04	4	1.96	4	1.68

**Table 7:** Sample effects for SCL change.

	SCL Change	
	Desc_Stairs	Desc_Ramp1
Young adults	mean = −3.69 sd = 3.40	mean = −0.76 sd = 4.41
Older adults	mean = −0.99 sd = 3.43	mean = 2.76 sd = 4.33
Mann-Witney	U = 86.00	U = 91.00

Columns represent the spaces for which there was a sample effect; rows represent the mean and standard deviations (sd) for each sample and the value of the non-parametric Mann-Witney test (U), respectively. Descending Stairs (Desc_Stairs), Descending Ramp (Desc_Ramp).

**Table 8:** Space effect for each physiological measure for each sample.

SCL Change		HR Change	
Older Adults	Young Adults	Older Adults	Young Adults
X^2^(6) = 16.97	X^2^(6) = 14.44	X^2^(6) = 12.15 (p=0.059)	No difference
NR1—NR2			
NR1—NR3			
NR1—Desc_Stairs	NR1—Desc_Stairs	NR1—Desc_Stairs	
NR1—Desc_Ramp2		NR1—Desc_Ramp1	
NR2—Desc_Ramp1			

The first row represents the value of the Chi Squared test for differences between all seven neutral and non-neutral spaces. The following rows represent pairs of spaces for which statistically significant differences were found. Descending Stairs (Desc_Stairs), Descending Ramp (Desc_Ramp), Ascending Ramp (Asc_Ramp), Neutral Room (NR).

**Table 9:** The effect of the CAVE in the sense of presence.

	SUS Scale	W&S Scale
	Sense of Presence	Awareness of Devices
Pocket CAVE	mdn = 3 sd = 1.21	mdn = 5 sd = 1.68
HLRS CAVE	mdn = 2 sd = 1.49	mdn = 4 sd = 1.94
Mann-Whitney	U = 245.5	U = 268.5

Columns represent the items of the scales for which there was a CAVE effect; rows represent the median (mdn) and standard deviations (sd) for each CAVE and the value of the non-parametric Mann-Witney test (U), respectively.

## Supplementary Materials

The following are available online at https://www.mdpi.com/1424-8220/19/11/2447/s1.
