# Supplementary material for: Physiological Arousal Quantifying Perception of Safe and Unsafe Virtual Environments by Older and Younger Adults
Source: Sensors (Basel). 2019 May 29;19(11):2447. doi: 10.3390/s19112447 (PMC6603542; doi:10.3390/s19112447)
Supplement: Supplementary file 1 [file sensors-19-02447-s001.pdf]

sensors

Article

Physiological Arousal Quantifying Perception of Safe and Unsafe Virtual environments by Older and Younger Adults

Sofia Leite [1], Miguel S. Dias [2,3], Sara Eloy [2,*], João Freitas [4], Sibila Marques [2], Tiago Pedro [2] and Lázaro Ourique [2]

1 CINTESIS—Center for Health Technology and Services Research, Faculty of Medicine, University of Porto, 4200-450 Porto, Portugal; sofiarsleite@gmail.com
2 Instituto Universitário de Lisboa (ISCTE-IUL), ISTAR-IUL; 1649-026 Lisboa, Portugal; sibila.marques@iscte-iul.p (M.S.D.); sibila.marques@iscte-iul.pt (S.M.); tiago.miguel.pedro@live.com.pt (T.P.); lazaro.ourique@gmail.com (L.O.)
3 ADENE, Agência para a Energia, 1050-065 Lisboa, Portugal
4 DefinedCrowd, 1500-399 Lisboa Portugal; jdcfreitas@live.com.pt
* Correspondence: sara.eloy@iscte-iul.pt; Tel.: +351-969-954-190

Received: 17 March 2019; Accepted: 25 May 2019; Published: date

Final questionnaires

Thank you for having participated in this experiment.

To finish we will ask you to answer a questionnaire, starting by reviewing the experience you had with the virtual environment.

We need your honest opinion regarding the building you just saw, so we can improve it in every way. There are no right or wrong answers and we are interested in the most immediate response regarding this building. To answer we ask that you think of the space you just experienced and responds to what extent you agree with the following statements on a scale 1-7 (where 1 = totally disagree and 7 = totally agree):

1. The space is beautiful

**Totally disagree**						**Totally agree**
**1**	**2**	**3**	**4**	**5**	**6**	**7**

2. The space is safe

**Totally disagree**						**Totally agree**
**1**	**2**	**3**	**4**	**5**	**6**	**7**

3. The space transmits fear

**Totally disagree**						**Totally agree**
**1**	**2**	**3**	**4**	**5**	**6**	**7**

4. The space is calm

Totally disagree						Totally agree
1	2	3	4	5	6	7

5. The space transmits anxiety

Totally disagree						Totally agree
1	2	3	4	5	6	7

We will now present you with some images of specific parts of the virtual environment. Please observe the images that the researcher will present you and answer to what extent you agree with the statements for each of the images on a scale 1-7 (where 1 = totally disagree and 7 = totally agree):

Image 1 (Figure 1)

1. The space is safe

Totally disagree						Totally agree
1	2	3	4	5	6	7

2. The space transmits fear

Totally disagree						Totally agree
1	2	3	4	5	6	7

3. The space transmits anxiety

Totally disagree						Totally agree
1	2	3	4	5	6	7

Image 2 (Figure 2)

1. The space is safe

Totally disagree						Totally agree
1	2	3	4	5	6	7

2. The space transmits fear

Totally disagree						Totally agree
1	2	3	4	5	6	7

3. The space transmits anxiety

Totally disagree						Totally agree
1	2	3	4	5	6	7

Image 3 (Figure 3)

1. The space is safe

Totally disagree						Totally agree
1	2	3	4	5	6	7

2. The space transmits fear

Totally disagree						Totally agree
1	2	3	4	5	6	7

3. The space transmits anxiety

Totally disagree						Totally agree
1	2	3	4	5	6	7

Image 4 (Figure 4)

1. The space is safe

Totally disagree						Totally agree
1	2	3	4	5	6	7

2. The space transmits fear

Totally disagree						Totally agree
1	2	3	4	5	6	7

3. The space transmits anxiety

Totally disagree						Totally agree
1	2	3	4	5	6	7

Image 5 (Figure 5)

1. The space is safe

Totally disagree						Totally agree
1	2	3	4	5	6	7

2. The space transmits fear

Totally disagree						Totally agree
1	2	3	4	5	6	7

3. The space transmits anxiety

Totally disagree						Totally agree
1	2	3	4	5	6	7

Now answer the following questions: (mark with an x your answer)

1. Rate your sense of being in the virtual environment, on a scale of 1 to 7, where 7 represents your normal experience of being in a place.

Look like being in the virtual world						Look like being in a place
1	2	3	4	5	6	7

2. To what extend were there times during the experience when the virtual environment was the reality for you? (1= never; 7= almost always)

Nowhere						Almost always
1	2	3	4	5	6	7

3. When you think back to the experience, do you think of the virtual environment more as images that you saw or more as somewhere that you visited?

Images I saw						somewhere I visited
1	2	3	4	5	6	7

4. During the time of the experience, what was the strongest on the whole, your sense of being in the virtual environment or of being elsewhere?

Being in the virtual environment						Being elsewhere
1	2	3	4	5	6	7

5. During the time of your experience, did you often think of yourself that you were actually in the virtual environment?

I was not in the virtual environment						I was in the virtual environment
1	2	3	4	5	6	7

6. How natural was the mechanism that controlled the through the environment (joystick and glasses)?

Extremely artificial			Borderline			Completely natural
1	2	3	4	5	6	7

7. How quickly did you adjust to the virtual environment experience?

Not at all			Slowly			Less than one minute
1	2	3	4	5	6	7

8. How aware were you of your display and control devices (joystick and glasses)?

Not aware at all			Mildly aware			Very aware
1	2	3	4	5	6	7

9. How distracting was the control mechanism (joystick and glasses)?

Not at all			Mildly distracting			Very distracting
1	2	3	4	5	6	7

10. Were you wearing glasses or contact lenses during the experiment?

☐ **Yes** ☐ **No**

11. How do you evaluate you heath condition in general? (mark with an "X" the number that corresponds to your answer)

Very bad			Medium			Very good
1	2	3	4	5	6	7

12. Circle how much each symptom below is affecting you right now:

1. General discomfort	None	Slight	Moderate	Severe
2. Fatigue	None	Slight	Moderate	Severe
3. Headache	None	Slight	Moderate	Severe
4. Eye strain	None	Slight	Moderate	Severe
5. Difficulty focusing	None	Slight	Moderate	Severe
6. Salivation increasing	None	Slight	Moderate	Severe
7. Sweating	None	Slight	Moderate	Severe
8. Nausea	None	Slight	Moderate	Severe
9. Difficulty concentrating	None	Slight	Moderate	Severe
10. "Fullness of the Head"	None	Slight	Moderate	Severe
11. Blurred vision	None	Slight	Moderate	Severe
12. Dizziness with eyes closed	None	Slight	Moderate	Severe
13. *Vertigo	None	Slight	Moderate	Severe
14. **Stomach awareness	None	Slight	Moderate	Severe
15. Burping	None	Slight	Moderate	Severe

* Vertigo is experienced as loss or orientation with respect to vertical upright

** Stomach awareness is usually used to indicate a feeling of discomfort which is just short of nausea
